# Sources of variation in cell-type RNA-Seq profiles

**DOI:** 10.1371/journal.pone.0239495

**Published:** 2020-09-21

**Authors:** Johan Gustafsson, Felix Held, Jonathan L. Robinson, Elias Björnson, Rebecka Jörnsten, Jens Nielsen

**Affiliations:** 1 Department of Biology and Biological Engineering, Chalmers University of Technology, Gothenburg, Sweden; 2 Wallenberg Center for Protein Research, Chalmers University of Technology, Gothenburg, Sweden; 3 Mathematical Sciences, University of Gothenburg and Chalmers University of Technology, Gothenburg, Sweden; 4 Department of Molecular and Clinical Medicine/Wallenberg Laboratory for Cardiovascular and Metabolic Research, University of Gothenburg, Gothenburg, Sweden; 5 BioInnovation Institute, Copenhagen, Denmark; John Curtin School of Medical Research, AUSTRALIA

## Abstract

Cell-type specific gene expression profiles are needed for many computational methods operating on bulk RNA-Seq samples, such as deconvolution of cell-type fractions and digital cytometry. However, the gene expression profile of a cell type can vary substantially due to both technical factors and biological differences in cell state and surroundings, reducing the efficacy of such methods. Here, we investigated which factors contribute most to this variation. We evaluated different normalization methods, quantified the variance explained by different factors, evaluated the effect on deconvolution of cell type fractions, and examined the differences between UMI-based single-cell RNA-Seq and bulk RNA-Seq. We investigated a collection of publicly available bulk and single-cell RNA-Seq datasets containing B and T cells, and found that the technical variation across laboratories is substantial, even for genes specifically selected for deconvolution, and this variation has a confounding effect on deconvolution. Tissue of origin is also a substantial factor, highlighting the challenge of using cell type profiles derived from blood with mixtures from other tissues. We also show that much of the differences between UMI-based single-cell and bulk RNA-Seq methods can be explained by the number of read duplicates per mRNA molecule in the single-cell sample. Our work shows the importance of either matching or correcting for technical factors when creating cell-type specific gene expression profiles that are to be used together with bulk samples.

## Introduction

RNA Sequencing is a well-established method for comparing the transcriptome between different cell types, conditions and cell states [1]. Cell types can be separated from samples, for example by using fluorescence-activated cell sorting (FACS) [2] or magnetic activated cell sorting (MACS) [3] before sequencing, and recent advances have made it possible to use RNA-Seq at the single-cell level and to sequence hundreds of thousands of cells [4]. The ever-growing collection of publicly available data enables integrative data analysis across many datasets, making it possible to discover system-wide phenomena. Such analyses are however made difficult by systematic batch effects across laboratories and technologies, posing a large challenge for data analysis.

Single-cell RNA-Seq facilitates the study of distinct cell types. However, the number of patients involved in such experiments is usually small compared to datasets containing bulk data from biopsies, such as The Cancer Genome Atlas (TCGA). It is therefore desirable to be able to conduct studies on bulk data with mixed cell types, with the help of mathematical tools that can help extract similar information as is available in single-cell data. One example of such a tool is cell type deconvolution, which estimates the fractions of different cell types in a mixed sample from the RNA-Seq data. This is implemented in for example CIBERSORTx [5], EPIC [6], CPM [7], MuSiC [8], and xCell [9] (although xCell is based on gene set enrichment analysis rather than deconvolution). Most implementations require gene expression profiles (GEPs) for each of the cell types into which the mixed sample is to be deconvolved. Some methods work with single-cell data [7, 8], others with a representative expression profile [6], but in general they need a representative expression of the cell types in the sample. Other tools that also need gene expression profiles for cell types include an extension of deconvolution often referred to as digital cytometry, which is implemented in CIBERSORTx [5], and automatic cell type annotation of cell types in single-cell data [10, 11].

Representative RNA-Seq gene expression profiles for cell types can be created from either FACS/MACS-sorted bulk samples or single cell datasets where the average expression of cell populations can be used. However, the RNA-seq profiles for a cell type can vary substantially, both due to biological differences between samples and technical biases. It is therefore challenging to construct universal gene expression profiles for cell types that perform well in all conditions. The variation in RNA-Seq originating from sequencing at different laboratories has previously been examined [12, 13]. In these studies, each laboratory has been given detailed sample preparation and sequencing instructions, enabling estimation of technical variation introduced from distinct technical factors. These studies, however, do not describe the typical case encountered for methods such as deconvolution of cell type fractions. In such cases, the cell type profiles are typically generated once and reused for many studies, often using different laboratory procedures, or even generated from single-cell data [5]. In addition, the cell type profiles and samples may be generated from different tissues. An example of such a case is using immune cell profiles from blood for estimation of cell type fractions in bulk tumor data from TCGA, which is made available by xCell [9]. Examples of generic cell type profiles generated for the purpose of deconvolution of cell type fractions are LM22 from CIBERSORT [14], IRIS [15, 16], and immunoStates [17].

There are a number of methods for measuring the impact of different factors, such as cell type, tissue of origin, or dataset on gene expression. ANOVA is commonly used to estimate such differences, for example. Another example is variancePartition [12], a method developed by Hoffman et al. that is based on mixed effects modeling and specifically adapted for RNA-Seq data. Hoffman et al. showed that variancePartition outperforms ANOVA for such analyses, supporting it as a strong candidate for the analysis of cell type profiles in the present study.

Normalization is a crucial step when analyzing RNA-Seq data. In the beginning of the RNA-Seq era, library size normalization, for example FPKM [18] and TPM [19], was commonly employed. It was later discovered that the library size is often strongly affected by a few highly expressed genes with stochastic behavior, which introduces a bias across samples. This shortcoming of library size normalization is remedied by methods such as the trimmed mean of M values (TMM) [20] and Relative Log Expression (RLE) in DESeq2 [21]. These methods are designed to operate directly on gene counts and work under the assumption that most genes are not differentially expressed across samples. The restriction to work on gene counts makes it more difficult to normalize single-cell data based on unique molecular identifiers (UMIs) together with bulk RNA-Seq. Bulk counts need to be normalized by transcript length, such as in FPKM and TPM normalization, to yield a representative gene expression, since longer transcripts will yield more mRNA fragments, which are all counted. On the other hand, droplet-based 3’ single-cell data, such as 10X Chromium data, should not be normalized by transcript length, since only the mRNA fragments closest to the polyA tail are counted. Consequently, the methods working on counts cannot directly account for transcript length, while library size normalization, which allows for such length correction, is sensitive to highly expressed noisy genes. An extension of TMM, GeTMM [22], addressed the issue of transcript length by dividing the counts for each gene by gene length before TMM normalization. An additional challenge with single-cell data is the high zero content, which causes problems for TMM and RLE normalization. In the case of cell type profiles, this issue can be remedied by simply pooling the transcriptomes of many cells before normalization. Alternatively, a deconvolution approach can be used to address this issue [23].

A common technique used to overcome technical biases is to computationally remove batch effects. The batch effect removal tool ComBat [24] implements a strategy where differences across batches in mean and dispersion of each gene are removed, regardless if the source of the variation is technical or biological. Another common method is to model the batch effect as a covariate in a generalized linear model, which is supported by for example edgeR [25] for differential expression. However, ComBat and similar tools require overlapping samples with similar biological traits across the datasets. This overlap does not exist between expression profiles of distinct cell types and biopsies containing a mix of cell types. CIBERSORTx employs a batch correction strategy using ComBat where mixed samples are created *in silico* by mixing the cell type expression profiles at different fractions. The batch correction applied on the synthetic mixed samples is then projected back to the cell types. A drawback of this approach is that it introduces a bias depending on the cell fractions selected in the synthetic mixture. Acquiring representative cell type profiles for a given dataset of mixed samples remains a challenge. In this study, we sought to quantify the relative importance of the most important factors that cause undesired variation between gene expression profiles of individual cell types for typical cases encountered in deconvolution and other methods that use cell type profiles. We assembled a collection of public datasets and evaluated normalization and batch correction methods, quantified different sources of variation between samples for different sets of genes, and investigated technical biases between single-cell and bulk RNA-Seq. In addition, we evaluated the impact of using different cell type profiles in deconvolution.

## Results

### Data preparation

To limit the complexity of the study, only B and T cell profiles were used. We gathered 74 publicly available RNA-Seq bulk samples of B and T cells from 5 different sources, and pooled 31 cell populations from single-cell data from 7 public datasets, for a total of 105 samples (Table 1, S1 Table). The data sources were chosen to span different laboratories and contain samples with different cell subtypes and tissues of origin. In addition, we investigated a dataset [26] containing both bulk and single-cell data from the same samples to examine biases between single-cell and bulk RNA-seq.

**Table 1:** List of RNA-Seq datasets used in this study.

ID	Description	Data Type	Source
HCA CB	Umbilical cord blood PBMCs from the Human Cell Atlas; in total ~254,000 cells from 8 patients.	Single cell, 10x genomics, UMI counts.	Li et al [38], Rozenblatt-Rosen et al [39]. The data can be downloaded from https://data.humancellatlas.org/, Census of immune cells.
LC	~39,000 cells from the tumor microenvironment of lung cancers and ~13,000 cells from adjacent healthy tissue. The cells originate from 5 patients.	Single cell, 10x genomics, UMI counts.	Lambrechts et al [40]. The data is available in in ArrayExpress under accessions E-MTAB-6149 and E-MTAB-6653.
PBMC68k	~68,000 PBMCs from blood, one patient.	Single cell, 10x genomics, UMI counts.	Zheng G.X.Y. et al [4]. The data is available at 10x Genomics’ home page.
B10k	~10,000 FACS-sorted CD19+ B cells from blood, one patient.	Single cell, 10x genomics, UMI counts.	Zheng G.X.Y. et al [4]. The data is available at 10x Genomics’ home page.
CD4TMEM	~10,000 FACS-sorted CD4+/CD45RO+ Memory T Cells, one patient.	Single cell, 10x genomics, UMI counts.	Zheng G.X.Y. et al [4]. The data is available at 10x Genomics’ home page.
TCD8	~10,000 FACS-sorted CD8+ T cells from the blood of a single patient.	Single cell, 10x genomics, UMI counts.	Chen et al [41]. The data is available for download on GEO data repository, accession number GSE 112845.
MEL	~4,600 cells from the tumor microenvironment of Melanoma, 19 patients.	Single cell, SMART-Seq2, TPM	Tirosh et al [42]. The data is available for download on GEO data repository, accession number GSE 72056.
EVAL	Dataset produced for evaluating the performance of existing single-cell technologies. Data from mouse brain, PBMC and cell lines. Data includes 7 single-cell technologies and bulk, all performed on the same samples.	Single cell data from 7 different technologies and corresponding bulk samples, counts/ UMI counts/ TPM	Ding et al [26]. The data is available for download at the Single Cell Portal, id SCP425.
BULK 1	In total 6 bulk samples from B cells of varying origin.	Bulk RNA-Seq, FASTQ files	The ENCODE Consortium [43, 44], Gingeras. The samples can be downloaded individually from ENCODE.
BULK 2	In total 7 bulk samples from B cells (1) and T cells (6) of varying origin.	Bulk RNA-Seq, FASTQ files	The ENCODE Consortium [43, 44], Stamatoyannopoulos and Weng. The samples can be downloaded individually from ENCODE.
BULK 3	In total 12 bulk samples from B cells (6) and T cells (6) of varying origin.	Bulk RNA-Seq, FASTQ files	The functional annotation of the mammalian genome 5 (FANTOM5) [45, 46]. The data can be downloaded from FANTOM5.
BULK 4	In total 39 bulk samples from B cells (16) and T cells (23) of varying origin.	Bulk RNA-Seq, FASTQ files	The BLUEPRINT Epigenome Project [47]. The samples can be downloaded individually from BLUEPRINT.
BULK 5	In total 10 PBMC bulk samples from B cells (5) and T cells (5).	Bulk RNA-Seq, RPKM/counts	Pabst et al [48], GSE 51984.

### Normalization and batch effects

First, we investigated data normalization approaches, which is challenging since our dataset contains both bulk data and pooled single-cell samples from UMI-based methods. This prevents the typical use of normalization methods that operate on counts, since the counts are not directly comparable between these sequencing technologies. In short, the bulk samples need to be corrected for transcript length, whereas the drop-seq based samples should not. We therefore decided to test three well-established methods that can operate on non-count data: library size normalization (TPM/CPM) [19], Trimmed Mean of M-values (TMM) [20], and quantile normalization [27]. TMM was originally designed to work on counts with a known library size; we therefore scaled the TPM values to pseudo-counts (Methods). To avoid the stochasticity from lowly expressed genes, we only analyzed genes with a mean expression of at least 1 TPM across all samples.

**Fig 1:**
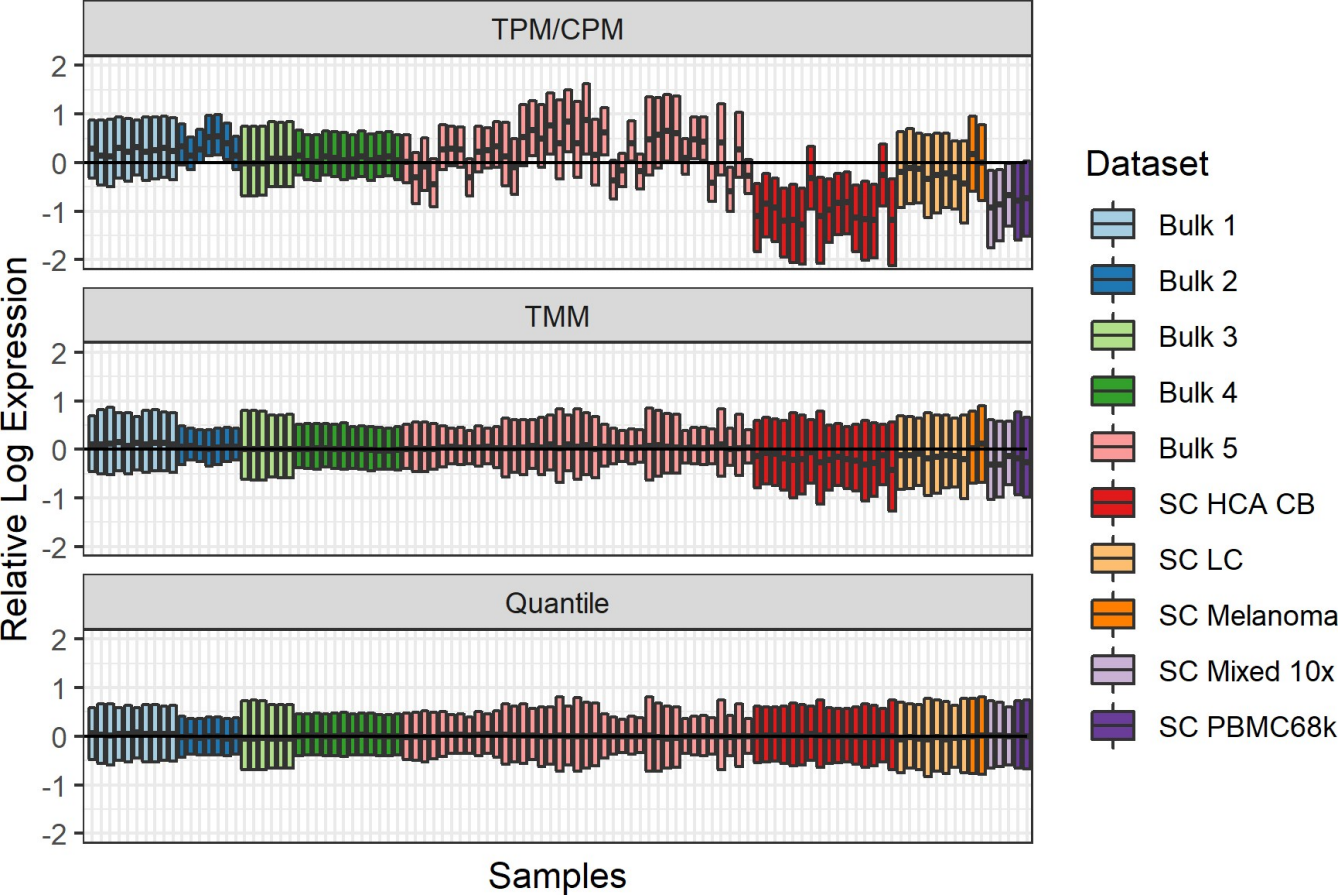
Evaluation of normalization methods for combined single-cell and bulk data. Each bar is a boxplot over all genes describing the log_2_ fold change in gene expression between the expression in the sample and the mean expression over all samples. All genes ≥ 1 TPM on average across all samples, in total 12,072 genes, were included for each sample.

**Fig 1** Comparing the relative log expression across all genes and samples for three normalization methods highlights the inadequacy of library size normalization for these samples (Fig 1). The drop-seq-based pooled single-cell samples (all single-cell samples except SC Melanoma) are especially problematic; a large portion of the genes are lowly expressed compared to the bulk samples. TPM normalization between bulk samples also fails to scale the samples properly, which has been shown previously [20]. TMM and quantile normalization succeed in overcoming most of the normalization issues, both in terms of mean and variation of the relative log expression. The advantage of TMM over quantile normalization is that it only scales the samples, minimizing the introduction of additional technical biases, while quantile normalization replaces all expression values. On the other hand, TMM assumes that most genes are not differentially expressed. This is a reasonable assumption here, but it may be inappropriate when comparing mixed bulk samples and GEPs for cell types. For the analyses in this study, we selected TMM as the normalization method unless otherwise noted.

**Fig 2:**
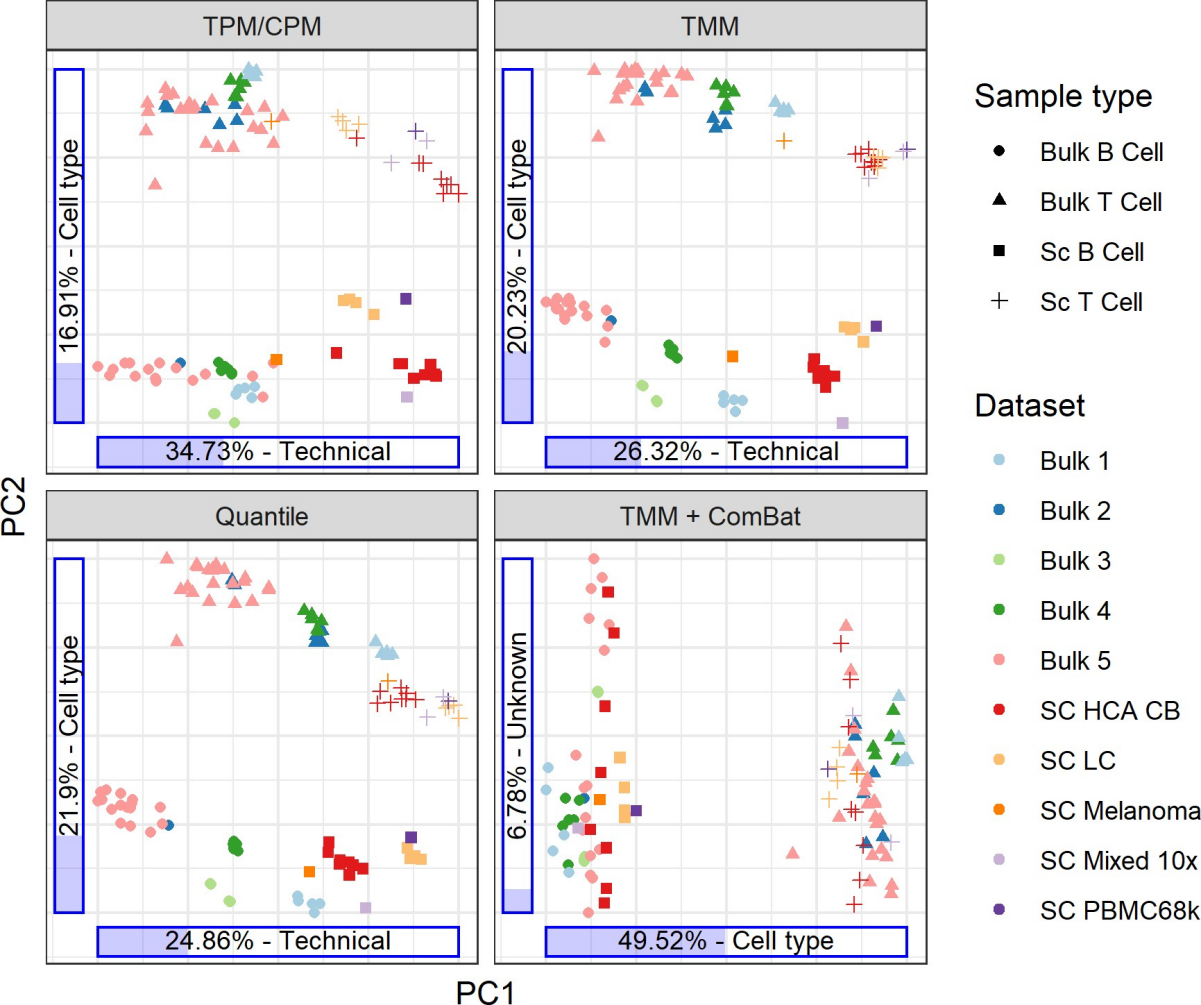
Visualization of normalization and batch effects using PCA. All genes ≥ 1 TPM on average across all samples, in total 12,072 genes, were included for each sample.

We used PCA to investigate the batch effects between different labs and between single-cell and bulk data. Fig 2 shows that without any batch correction, samples group by dataset, and the first principal component mainly describes technical variation between datasets. The drop-seq-based 10x datasets tend to cluster together, and the same holds for the bulk samples. The only Smart-Seq2 single-cell data present (SC Melanoma) seem to be more similar to bulk than the other single-cell samples. The normalization method clearly matters; the first component explains less of the variation with a better normalization method, and the batch effects are more pronounced due to the reduced technical noise. We applied ComBat [24] from the sva [28] R package to remove batch effects between datasets, with the instructions to preserve cell type differences. ComBat effectively removes all systematic differences in mean and dispersion between datasets, except for those specified in the model matrix (cell type in our case), however it does not distinguish between biological and technical variation. Applying Combat will thus remove any biological differences across datasets not specified in the model matrix, such as in our case differences in cell subtype and tissue distribution between the datasets, which may affect downstream analysis. We did not include cell subtype and tissue in the design matrix since these properties are represented by too few samples. To illustrate that biological effects may be lost, we used ComBat without specifying cell type in the design matrix followed by PCA (S1 Fig). Since the Bulk 3 dataset only contains B cells, ComBat interprets that samples in this dataset on average look more like B cells. ComBat incorrectly compensates for this effect, placing these samples between the B and T cells of the other datasets along the PC1 axis (cell type).

### Sources of variation between samples

To quantify sources of variation in gene expression across samples we investigated to what extent different factors could explain the variance in gene expression. Factors examined were laboratory (which here includes different laboratory procedures and sequencing methods), UMI-based single-cell vs bulk, cell type, cell subtype (distinguishing between different types of B and T cells), tissue of origin (distinguishing between samples containing the same cell types but being collected from different tissues), individual, and whether samples are technical replicates. We normalized the 105 samples using TMM as described above and used the R package variancePartition [12] to estimate the importance of the factors to be investigated for each gene.

**Fig 3:**
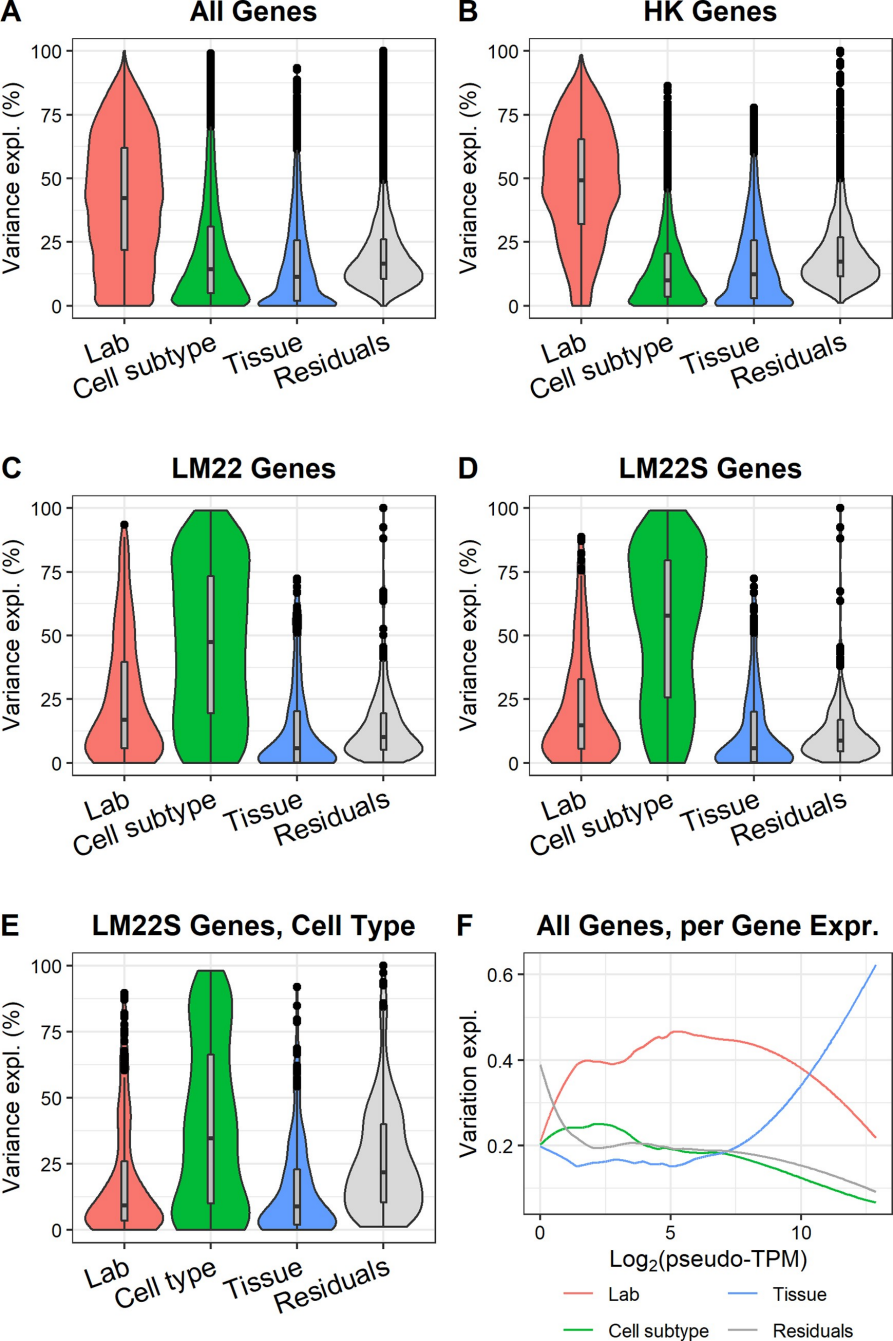
Explained variance in gene expression for bulk RNA-seq samples. A. All genes (12072 genes). B. Housekeeping genes (3393 genes). C. LM22 (395 genes). D. LM22S genes. E. Identical to D, with the difference that cell subtype is replaced with cell type (B/T). F. Explained variance per gene expression. The plot shows how the explained variance by the different factors change with gene expression (Loess fit, span = 0.3).

Fig 3 shows the variation induced by the different factors across bulk samples, investigating the factors laboratory, cell type, cell subtype, and tissue of origin. Fig 3A shows the explained variance for factors when including all genes. Fig 3B–3D show how the explained variance changes for different gene sets: housekeeping genes, genes used in deconvolution of immune cells (the genes selected for deconvolution when using the LM22 cell type profiles, developed by the CIBERSORT [29] team), and a subset of LM22 genes that exhibit substantial expression differences between B and T cells (absolute log_2_ fold change > 1, henceforth named LM22S). When including all genes, lab is the dominating factor, highlighting the importance of working with a reduced gene set for methods such as deconvolution. For housekeeping genes, defined as genes having a stable expression across tissues and cell types [30], lab explains more of the variance compared to the case where all genes were used, which is consistent with the expectation of lower biological variation in this gene set. As expected, cell subtype is the most important factor for explaining variance among the LM22 genes (Fig 3C), and even more so for the genes in the LM22S subset (Fig 3D). Interestingly, cell subtype still only explains roughly half of the variation in the LM22S gene set, posing a challenge for deconvolution and similar methods. Fig 3E shows the explained variances for the LM22S genes if cell type is used as a factor instead of cell subtype. The lower explained variance indicates that many of the LM22S genes differ in expression between cell subtypes. This result is consistent with the formulation of the LM22 gene set (which LM22S is derived from), which was optimized for separating immune cell subtypes including those of T and B cells. To verify that the results were not dominated by outlier samples, we repeated the analysis for bulk samples excluding one sample each iteration. The maximum change for any factor was less than 2 percentage points, suggesting a negligible outlier effect.

To investigate the influence of gene expression on explained variance, we estimated explained variance as a function of gene expression for all genes (Fig 3F). As expected, the residuals explain more of the variance for lowly expressed genes, since sampling noise lead to higher variation for such genes. Although lab remains the dominant factor for much of the gene expression range, tissue becomes more important for highly expressed genes. The same analysis was performed for the LM22 genes only, which as expected yielded a higher importance for cell subtype, while tissue was less important (S2 Fig). The increasing importance of tissue with gene expression was not observed for these genes, suggesting that this effect is of less importance for applications such as deconvolution of cell type fractions.

To evaluate the effect of batch correction using ComBat, we performed the same analysis as in Fig 3 for batch-corrected data (S3 Fig). The lab factor now had very little influence on the variation, while cell type became more important and tissue approximately retained its importance. This effect is expected from the batch correction performed, where the goal was to remove differences across datasets and preserve differences across cell types. We also investigated the impact of using quantile normalization instead of TMM by comparing the explained variance of factors for these two methods across all samples (S4A and S4B Fig). Although lab explains slightly less variation for quantile normalization, the difference is small.

We also investigated the variance reduction from using samples from the same individual (taken at different time points) and technical replicates (the same sample sequenced multiple times) (S4E–S4H Fig). Our results indicate that technical replicates, although here represented by only a few samples, exhibit lower variance in gene expression than different biological samples. Samples from the same individual taken at different time points however show similar variance compared to samples taken from different individuals, which is contradictory to previous results indicating the importance of individuals for explaining variance [12]. In that study, however, the samples from the same individual were technical replicates, which show a lower variation in our measurements as well. Given the small number of samples, our results here should be interpreted with caution, and further studies are needed to fully characterize this source of variation.

**Fig 4:**
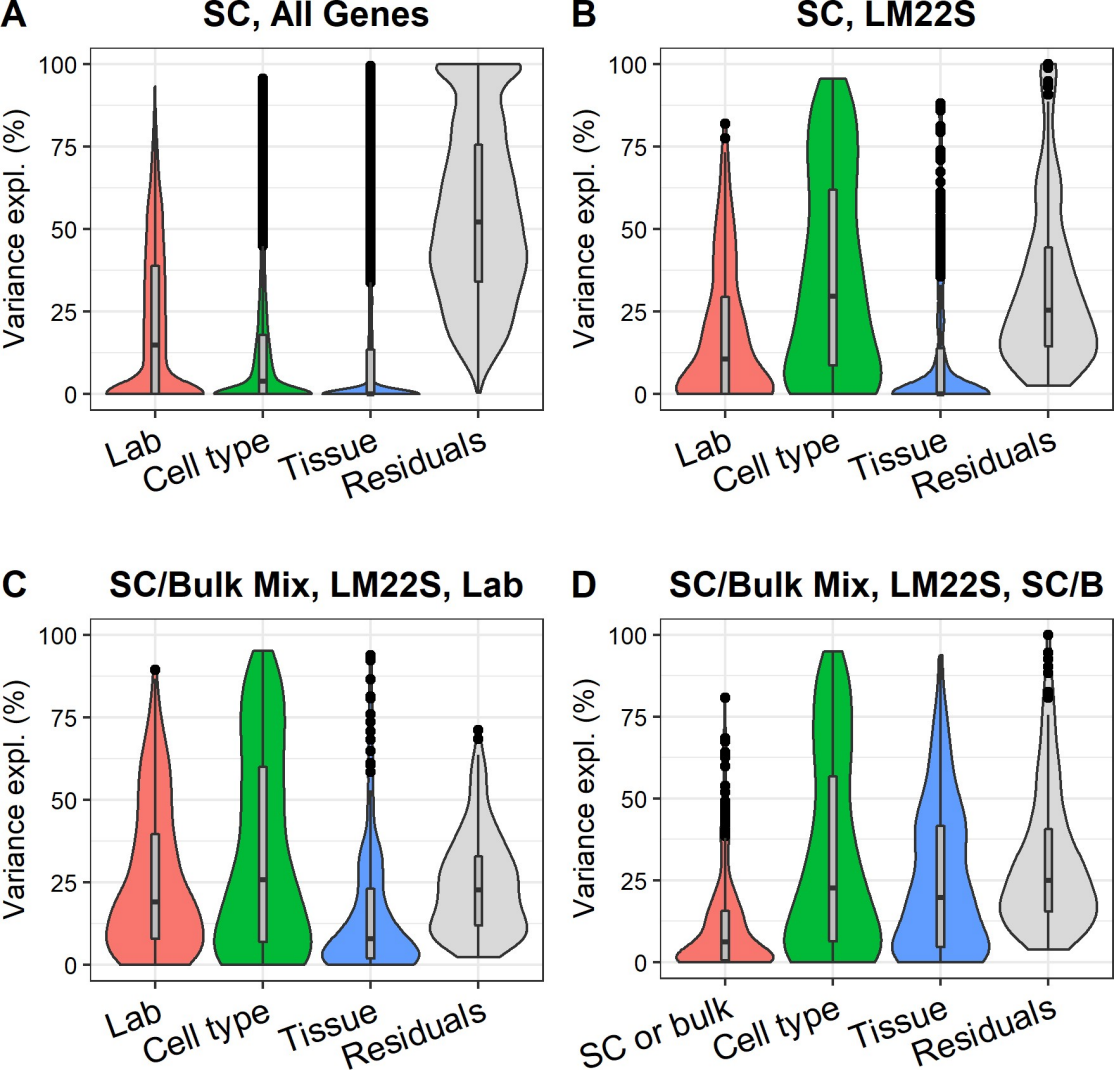
Explained variance for single-cell samples. A. Only single-cell samples, all genes. B. Only single-cell samples, LM22S. C. Mix of single-cell and bulk samples, LM22S. D. Identical to C, with the exception that lab is replaced by a flag indicating if the samples are single-cell or bulk.

To investigate the factors explaining the variance between profiles from pooled single-cell data, we used variancePartition on the single-cell samples to analyze the factors lab, cell type, and tissue. The SC melanoma dataset was excluded from the analysis because it was the only dataset using the Smart-Seq2 technology, and consequently had a high impact on the variance explained by lab (S4C and S4D Fig). We evaluated the effect of outliers the same way as described above for the bulk samples, and similarly found that the maximum change for any factors obtained by removing any one sample was less than 2 percentage points. Fig 4A and 4B show the explained variance when including all genes or LM22S genes, respectively. The fraction of unexplained variance (residuals) is larger for single-cell than bulk, indicating that there is much variation that we fail to model, and it is therefore likely more challenging to work with single-cell data for deconvolution and other computational methods that require cell type profiles. Potential sources of the increased unexplained variation include higher technical noise in single-cell data and the presence of misclassified cells. Fig 4C and 4D shows the explained variance in LM22S across all samples, both bulk and single-cell (still excluding the Smart-Seq2 samples). Specifically, the figures highlight the difference in explained variance between lab (where each lab is modeled separately) or by just separating single-cell and bulk samples (SC/B). Although there seem to be systematic differences in gene expression between single-cell and bulk, a large part of the variance is still explained by lab-specific factors.

### Differences between single-cell and bulk

To unravel the sources of variation between pooled single-cell data and bulk, we investigated the EVAL dataset which contains bulk and single-cell (10x) data generated from the same samples [26]. We used the cortex 1 and cortex 2 samples, originating from mouse brain, to determine if it was possible to identify technology-driven differences between 10x data and bulk. We first pooled the single cells for each cortex and normalized them together with the bulk data using TMM (FPKM data and UMI counts, as described earlier). A small set (232) of outlier genes were filtered (Methods).

**Fig 5:**
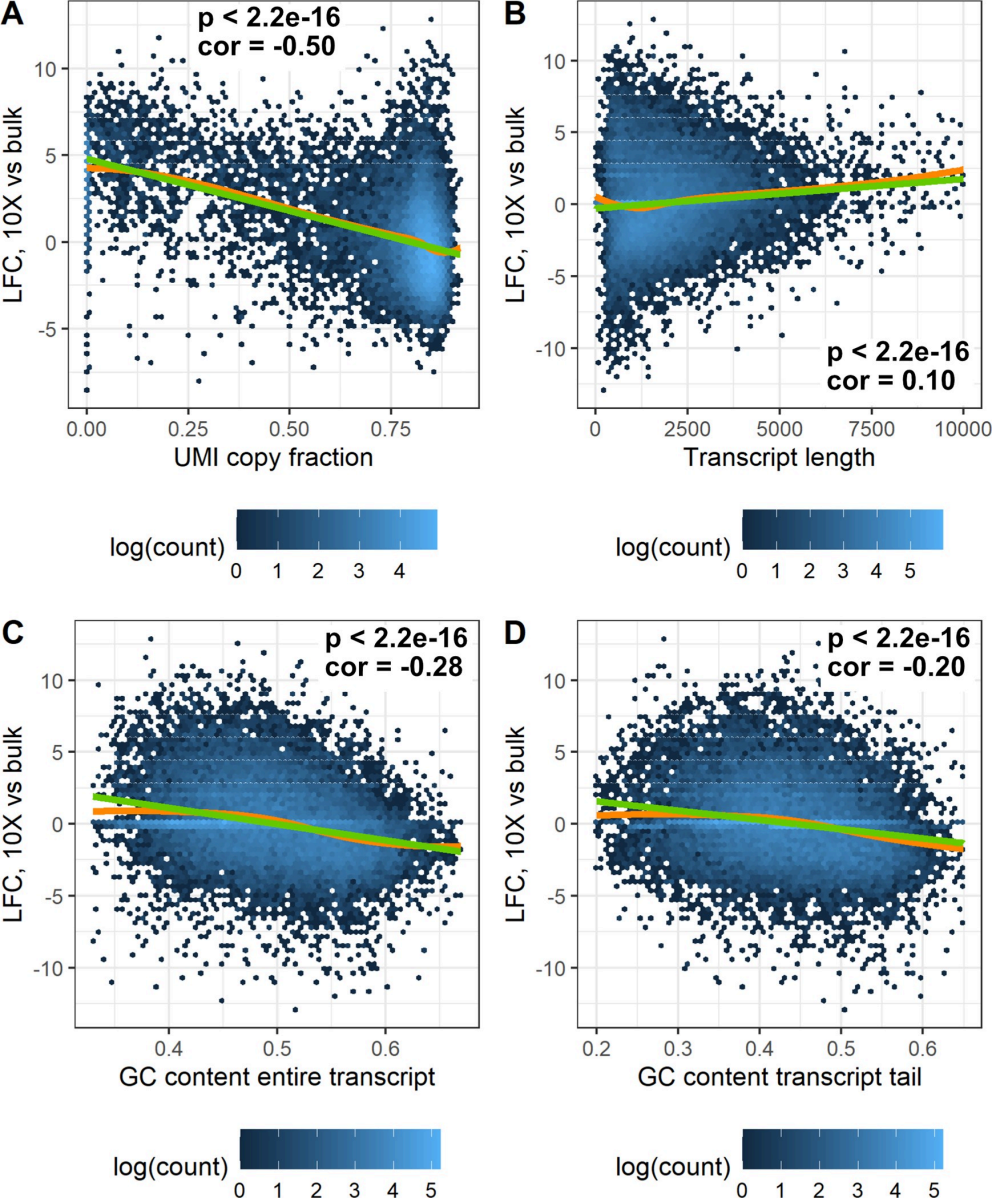
Log2 fold change between 10x data and bulk for each gene, plotted as a function of different covariates. The green line represents a linear fit, whereas the orange line shows a Loess fit. The data shown is from cortex 1 of the EVAL dataset. Each plot shows the p value for the linear fit (F-test) and the Pearson correlation. A. UMI copy fraction. Only genes with more than 5 molecules available for calculating UMICF are shown. B. Transcript length. C. The GC content of the entire transcript. D. The GC content of the 150 bases closest to the transcript tail.

We defined difference in gene expression per gene as the log2 fold change between the 10x pool and bulk data (TMM normalized as described above) and investigated to what extent that difference could be explained by different technical covariates across genes. First, we calculated the number of discarded UMI duplicates per UMI for each gene. We defined the UMI copy fraction as

$$UMICF=\frac{total\;counts-UMI\;counts}{total\;counts}$$

for each gene, representing the fraction of the counts that are filtered as UMI duplicates. Fig 5A shows a clear negative correlation (-0.50, Pearson correlation) between the difference in gene expression and UMICF (p < 2.2 * 10^-16, F Test). There could be several explanations for this effect. The covariate could represent differences in PCR amplification between genes. We reason that although such biases are mostly removed in the 10x data due to discarding of UMI duplicates, they are present in the bulk data, and the PCR amplification could be similar for the same gene in both cases. An alternative explanation could be that copies of the same molecules are assigned to different genes with similar sequence, such that they are counted as different molecules in the single-cell data. Such an effect would increase the total count for genes sharing copies of the same original molecule. A third possible reason for the negative slope could be that for some genes more reads are discarded due to alignment failure or quality filtering. Since there are often several copies per UMI, such an effect would be limited in the single-cell data but would have a large impact for bulk gene quantification.

Second, we investigated if transcript length introduces a bias due to differences in the sequencing protocol between 10x and bulk. Bulk reads can come from the entire transcript whereas the 10x reads originate from the sequences close to the polyA tail and is thereby less affected by transcript length. Fig 5B indicates that this covariate introduces a bias, although modest (Pearson correlation = 0.10), where longer transcripts in general seem to be over-penalized by the division of gene length in bulk (p < 2.2 * 10^-16, F Test).

Third, we investigated the effect of the GC content of genes since this is a known source of bias in RNA-Seq [31]. We investigated two covariates, the GC content of the entire transcript (Fig 5C) and the GC content of the 150 base pairs closest to the polyA tail (Fig 5D), to see if those better could explain the variability between 10x data and bulk. The transcript tail was investigated since only the mRNA fragment closest to the tail is used in the quantification of the 10x single-cell data, while all transcript fragments are quantified for bulk. Differences in GC content between the tail and the rest of the transcript could therefore theoretically introduce a bias between 10x single-cell data and bulk. Fig 5C and 5D shows that a higher GC content in general gives a higher expression in bulk compared to 10x (p < 2.2 * 10^-16 for both covariates, F Test), which could be related to PCR amplification biases.

**Fig 6:**
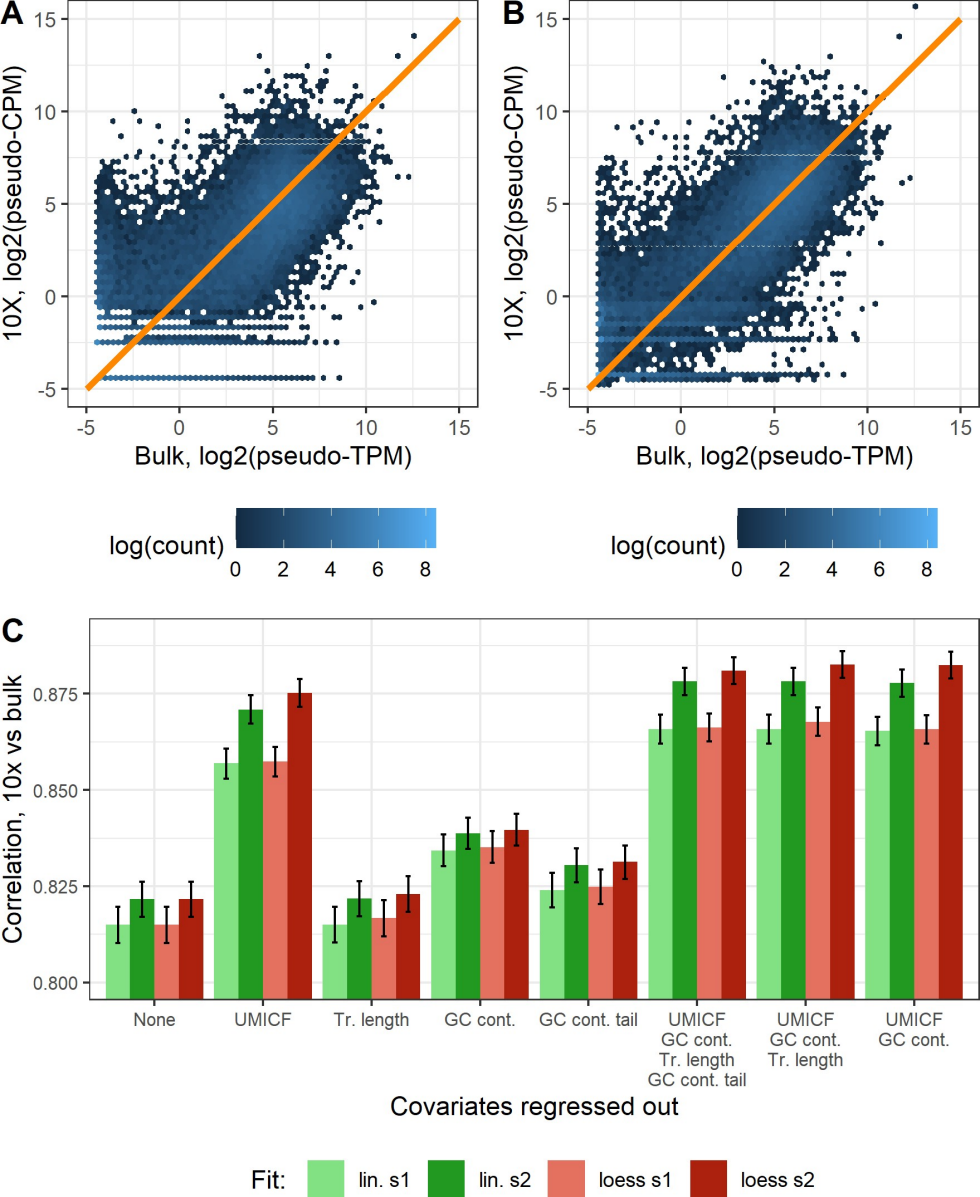
Improvement in correlation between 10x Chromium and bulk from regressing out covariates. A. Gene expression for cortex 1 from the EVAL dataset plotted as 10x vs bulk. The orange line represents a perfect correlation. B. Gene expression for cortex 1 from the EVAL dataset after regressing out the differences in UMICF and GC content between 10x and bulk using a loess fit, which improves the correlation. C. Average Pearson correlation coefficient between 10x data and bulk in log scale after regressing out technical covariates (UMI copy fraction, transcript length, GC content and GC content tail), using linear or loess regression. The correlation shown is the average of the correlations from cortex 1 and 2 of the EVAL dataset. The error bars represent the confidence interval, based on bootstrapping of genes to include in the correlation.

To evaluate how much of the differences between 10x data and bulk can be explained by the technical covariates, we measured the improvement in correlation between 10x and bulk data after regressing out the covariates. To exclude the possibility that the differences originate from stochasticity, i.e. lack of reproducibility of data, we first checked the log space correlation between cortex 1 and 2. Although the samples originate from slightly different parts of the brain, the different samples had a Pearson correlation of 0.989 for bulk and 0.981 for 10x, showing low stochasticity. Fig 6 shows the correlation improvement after regressing out different combinations of covariates, using both a linear and loess fit. It is evident that regressing out the UMICF covariate increases the correlation more than the other covariates (p < 2.2 * 10^-16, both Cortex 1 and 2, both loess and linear), though the other covariates explain some variation on their own. When all covariates are combined, the tail GC content does not add information (the correlation is nearly identical) and transcript length has a neglectable effect on correlation improvement (mean correlation improvement is 7.7 * 10^-4, although significant, p < 2.2 * 10^-16). The negligible effect of the GC content tail covariate could potentially be explained by a reduction in PCR bias due to UMI-collapsing, and because the first out of two amplification steps in the 10X Chromium protocol is conducted on full length transcripts. We conclude that the combination of UMICF and GC content is a good choice (significant improvement vs UMICF alone, p < 2.2 * 10^-16). The differences between regressing out a loess or linear fit for a covariate are generally small, although loess performs slightly better.

We also investigated the role of sampling effects when regressing out the UMICF variable (S1 Note, S5 and S6 Figs), concluding that sampling effects likely do not explain most of the correlation increase obtained from regressing out the UMICF variable.

### Deconvolution of cell type fractions

**Fig 7:**
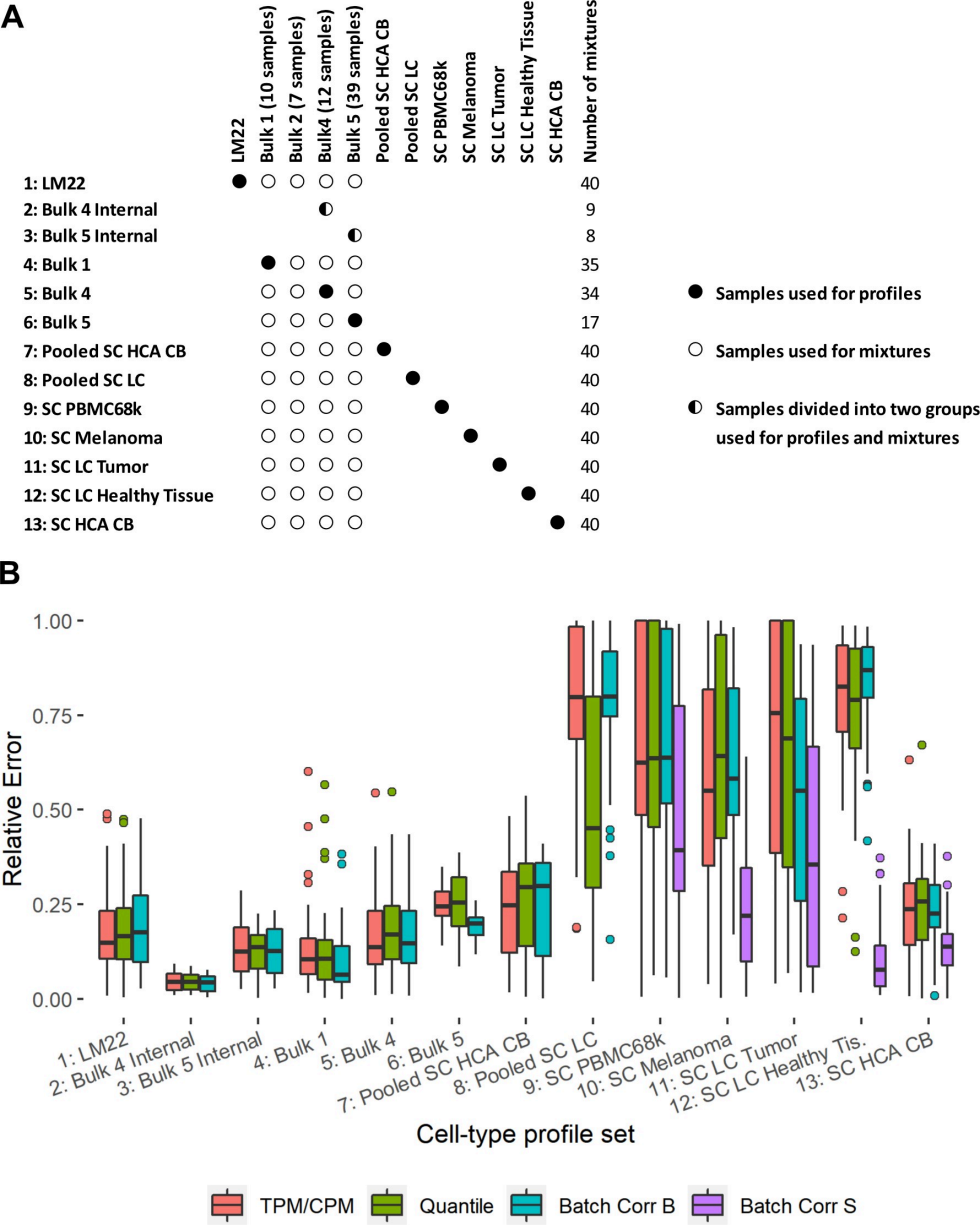
Deconvolution performance for different cell type profiles tested against in-silico generated bulk mixtures with 50% T cells and 50% B cells. A. Overview of the profiles and mixtures used for deconvolution. The rows represent deconvolution runs and the columns data sources. B. Deconvolution performance. The figure shows the relative error of the B cell fraction estimated by CIBERSORTx. The Bulk 4/5 Internal profiles were generated from the Bulk 4/5 lab and were tested against mixtures generated from other samples from the same lab. The remaining profiles were tested against 40 mixtures generated from Bulk 1, 2, 4, and 5 (Bulk 3 has no T cell samples).

To assess the impact of technical sources of variation on deconvolution of cell type fractions, we conducted a qualitative comparison of deconvolution performance across profiles generated from different datasets. We generated in total 40 synthetic mixed bulk samples containing 50% from a B cell sample and 50% from a T cell sample, both produced at the same lab. We further generated in total 12 pairs of cell type profiles (B cells and T cells from the same lab), originating from both single-cell and bulk data. Fig 7 shows the deconvolution performance from CIBERSORTx, which varies largely across profiles. As expected, the best results are obtained when using profiles and in-silico mixtures from the same lab, with no difference in tissue of origin or cell subtypes. The general conclusion that can be drawn is that the uncertainty in deconvolution performance is large unless the profiles and mixtures are generated under similar conditions, using similar technologies and laboratory procedures. Our results also suggest that using cell type profiles developed from single-cell data is more challenging. Furthermore, the performance varies much between sets of cell type profiles, where profiles generated from some datasets, such as HCA CB, performs much better than others (e.g. profiles generated from PBMC68k). Some of the single-cell profiles come from different tissues, which could partly explain the reduced deconvolution performance in those cases. However, the SC PBMC68k profiles originate from blood and contain a mix of cell subtypes. The lower performance for those profiles as compared to bulk profiles from similar cells (e.g. Bulk 4) can thus not be attributed to differences in tissue of origin or cell subtype only. CIBERSORTx supports two batch correction methods to remedy such issues; B-mode for cell type profiles from bulk and S-mode for single-cell profiles. We found that the S-mode batch correction seems to improve the performance considerably for single-cell profiles, while the B mode batch correction has a less pronounced effect.

## Discussion

Cell-type specific gene expression profiles are useful for analyzing bulk RNA-Seq samples containing mixed cell types, since it enables the use of advanced computational methods such as deconvolution of cell type fractions and digital cytometry. In this study, we investigated the impact of different sources of variation on cell-type specific gene expression profiles. We evaluated normalization methods and the effect of batch correction and used variancePartition to quantify the contribution of variance originating from differences in lab, cell-type, cell subtype and tissue of origin for different gene sets. Furthermore, we investigated the biases between UMI-based single-cell and bulk RNA-Seq. Finally, we examined the effect of cell type profile features on the performance of deconvoluting cell type fractions.

We found that the variance explained by different factors is heavily dependent on the gene set, where lab explains the most variance when considering all genes. For deconvolution of cell type fractions and similar methods, the genes that separate cell types are of greater interest. Although cell type/cell subtype is the factor that explains most variance here, other factors such as lab and tissue of origin also have a substantial effect on gene expression for these genes. Consistent with our findings, large variation across experiments together with small differences between some cell subtypes has previously been shown [32]. Our deconvolution results similarly show that indeed the performance of deconvolution decreases substantially when cell type profiles and mixed samples originate from different labs, and even more so if the profiles are generated from single-cell data. Hoffman et al has previously shown that the variance explained by lab is low [12]. In that study, however, they used data where all laboratories were given precise instructions on how to perform the experiment. Such an experiment does not reflect the usual use case for deconvolution and other such methods; that the cell profiles are generated beforehand using one method, and that the mixtures are generated by another lab, potentially using other protocols, or by using a different technology. Our results show that for the typical use case, the technical factors are substantial.

Normalization and batch correction are important steps in the analysis of RNA-Seq data. In this study, we have shown that while library size normalization is inadequate, TMM [20] applied to pseudo counts and quantile normalization [27] both work well for normalizing between bulk and single-cell data. T and B cells are both immune cell types; for more diverse cell types, more advanced normalization methods such as smooth quantile normalization [33], implemented for example in YARN [34], may be a useful approach since it can handle differences in gene expression distribution across different types of samples. We also show that ComBat [24] effectively removes technical batch effects. There are also other methods for batch correction, for example to model the batch effect as a covariate in a generalized linear model; this method is commonly used for differential expression analysis, for example in edgeR [25]. Such an approach would likely yield similar results as ComBat. These normalization and batch correction methods are limited in that they either assume that most genes are not differentially expressed or require some biological overlap across samples. Neither of these criteria are generally fulfilled when normalizing and correcting bulk data from mixed samples with cell-type specific gene expression profiles. The major challenge for batch correction is likely thus about how to batch correct samples containing mixtures of cell types against the cell type profiles, while selection of batch correction method, for example ComBat or GLM, is less important. CIBERSORTx [5] addresses this issue by implementing a method for using ComBat with in-silico mixtures of cell type profiles together with mixed bulk samples (S-Mode batch correction) which clearly improves the deconvolution performance, but there are still challenges remaining to obtain accurate deconvolution results when the technical platforms are not matched.

Our strategy for TMM normalization is very similar to that of GeTMM [22], where counts are divided by gene length before calculating TMM. However, we see two advantages with our approach: 1) Although it is impossible to produce the correct number of counts for each gene for statistical calculations, we reason that our approach, where the total number of counts are preserved, will give a closer estimate than division by gene length. 2) By using TPM, where the transcript length is accounted for at transcript level rather than gene level, the length of each transcript is better corrected for. At gene level, a single length needs to be used for all transcripts, which may bias the calculation depending on differences in expressed splice variants of different lengths.

We also sought to examine in detail the technical biases present for cell-type specific gene expression profiles derived from UMI-based single-cell RNA-Seq when compared to bulk RNA-Seq data. We found that the number of duplicate reads per mRNA molecule in the single-cell data (UMICF) can explain a substantial fraction of the differences between single-cell and bulk, whereas transcript length biases are generally small in comparison to other effects. GC content has previously been reported to introduce bias [35], and it is likely that, to a large extent, this bias is caused by PCR [31]. We show that the UMICF covariate can explain more of this bias than GC content, and that these effects are partly correlated, suggesting that they at least partly describe the same underlying phenomenon. However, the combination of both covariates explains more of the variation between single-cell and bulk. A potential explanation is that GC content also provides information for genes with few reads for which duplicate reads per mRNA molecule is poorly estimated, or that GC content can explain other technical effects that are not captured by UMICF. It is likely that the true PCR effect in bulk is larger than what can be measured with the UMICF covariate; the PCR effect per gene is most likely different between 10x and bulk, since the amplification process is different. Although the UMICF covariate apparently is useful for estimating the PCR amplification in bulk, regressing out the true effect would most likely give a larger improvement in correlation between single-cell and bulk. Furthermore, we have recently shown that UMI collapsing does not fully remove PCR effects in single-cell [36], suggesting that the true PCR effect in bulk is larger than the difference in amplification between UMI-collapsed single-cell data and bulk. These results highlight the need for methods to compensate for PCR effects in bulk, perhaps by utilizing unique molecular identifiers or by using other methods to estimate PCR amplification per gene. In addition to the covariates we have examined, there are many other technical biases contributing to differences between single cell and bulk that could be investigated, such as differences in processing pipelines (in this case RSEM [37] for bulk vs Scumi [26] for single-cell), where UMIs mapped to multiple genes are discarded in the single-cell pipeline.

This study is limited in that we only investigated two cell types. We can thus not claim that the technical variation is of the same magnitude as differences across cell types in general, but only between B and T cells. Furthermore, the study was not fully balanced; some sources of variation are represented more strongly than others, which can have a slight effect on the results. For example, most samples originate from blood, increasing the influence of that specific environment when estimating the importance of the tissue of origin variation factor. In addition to these limitations, we only investigated one deconvolution software since the focus of this study was cell type profiles and not deconvolution algorithms; it could be that other methods are less sensitive to batch effects between profiles and mixtures.

Our work suggests that estimating the number of duplicate reads per mRNA molecule can help in predicting and correcting for technical bias and thereby yield more comparable samples, both across bulk samples, single-cell samples and between bulk and single-cell. These results need to be further validated in more datasets, and the factors introducing this bias need to be investigated in more detail. Although such factors may differ across experiments, it is possible that a library of factor patterns aggregated from many single-cell experiments could be used for a more generalized prediction and correction of bias in bulk data. Such a method would be useful for a broad range of applications extending beyond the generation of gene expression profiles for deconvolution or digital cytometry.

## Conclusions

In this study, we investigated the sources of variation in cell-type specific gene expression profiles. We demonstrated that technical effects resulting from different laboratory procedures and data types explains much of the variance across samples and confounds analyses such as deconvolution, but also that biological traits such as cell subtype and tissue of origin are important to consider when generating cell-type specific gene expression profiles. These results provide valuable insight to users of computational methods such as deconvolution of cell type profiles and digital cytometry, highlighting the importance of matching both technical protocols and biological traits between cell type profiles and bulk data samples.

## Methods

### Data preparation

We downloaded the publicly available RNA-Seq datasets listed in

Table 1, in total 74 bulk samples of B and T cells in addition to 8 single-cell datasets. The bulk B and T cell samples have different composition; some samples contain a mix of all cell subtypes of either B or T cells, while others contain specific cell subtypes (8 different subtypes in total for T cells, 3 for B cells).

We downloaded fastq files for BULK 1–4 to reduce the technical variability across datasets induced by the computational pipeline, and processed them using Kallisto [49] (v. 0.45.0). We pseudo-aligned to the HG38 (version GRCh38.p12) genome with the parameters “kallisto quant -i transcripts.gtf.gz -o [output folder] -b 1 [fastq file 1] [fastq file 2]”. For BULK 5, we did not have access to fastq files and instead used the RPKM expression values produced by the authors, converted to TPM.

For single-cell datasets, cell type classifications were retrieved from the authors of the study in cases where it was not publicly available. For Smart-Seq2 data (the MEL dataset), we used the TPM values produced by the authors, and pooled the cells within a cell population by calculating the average expression per gene. For 10x data, we pooled the cells by first summing the counts from all cells for each gene, and then scaled the expression to a total sum of 10^6^ for all genes. For simplicity, only genes that existed in all datasets and could be properly converted to HGNC symbols were used in this study. The datasets B10k, CD4TMEM and TCD8 were treated as if they had been produced in the same laboratory (called “SC Mixed 10x data”) even though they had not, which was motivated by their use of similar techniques and too low sample numbers to be treated as separate labs.

All samples are described in more detail in S1 Table, including the design matrix and detailed information of each sample, for example cell subtype, tissue of origin and the number of cells in each pooled single-cell sample.

### Normalization, PCA and batch correction

TPM normalization was performed according to

$${TPM}_{i}=\frac{{10^{6}*E}_{i}}{\sum_{j}E_{j}},$$

where ***TPM***_***i***_ represents the normalized expression for gene ***i*** and ***E***_***x***_ is the expression of gene ***x*** before normalization.

TMM normalization was performed using the calcNormFactors [20] function in the edgeR package [25] (version 3.26.7). For pooled 3’ single-cell data TMM was applied directly to the pooled data. For bulk data, the following procedure was used: TMM was originally designed to work on counts, and needs to know the library size, but can work with non-integer data. Before applying TMM, the TPM values were scaled to pseudo-counts (*PC*_*i*_), where the sum of all gene expression values equals the original library size, according to

$${PC}_{i}={TPM}_{i}\frac{\sum_{j}{OC}_{j}}{\sum_{j}{TPM}_{j}},$$

where OC_x_ is the original counts for gene x. The pseudo-counts differ from the original counts in that they are corrected for transcript length, but with identical library size. The purpose of this scaling was to make the number of counts in the TMM normalization as similar as possible to the original counts, since this number is used in the normalization calculations. The same procedure should ideally be applied to Smart-Seq2 data, but since we didn’t have access to raw counts, TMM normalization was applied directly to TPM values for these two samples.

For quantile normalization we used the function normalize.quantiles in the preprocessCore package [50] (version 1.46.0).

PCA was performed using the R function prcomp with the parameter scale set to FALSE.

For batch correction we used the ComBat function in the SVA package, specifying that differences related to cell type should be preserved (in the model.matrix, using “~1 + cellType”). As batch, we used dataset id with one modification; the datasets PBMC68k, B10k and CD4TMEM were treated as the same dataset since they had to few samples to be batch corrected separately. We deemed that this was reasonable, since the data is produced in a similar way and published in the same publication.

### Log transformation

We applied log transformation for many analyses to make the expression data more normally distributed. The log transform was applied according to ***L_i_ = log*2(*E_i_+b*)**, where ***L***_***i***_ is the log transformed expression of gene ***i***, ***E***_***i***_ is the expression of gene ***i*** in pseudo-TPM, and ***b*** is a constant set to 1, which is added to avoid taking the logarithm of zero values.

We use the term “log_2_ fold change” (LFC) throughout the results to compare the expression of a gene between two samples. This was calculated as

$${LFC}_{i}={log}_{2}(\frac{E_{i,1}+b}{E_{i,2}+b}),$$

where ***E***_***i*,*1***_ and ***E***_***i*,*2***_ represent the expression of gene ***i*** in the two samples which are to be compared.

### Measuring the explained variance of factors

We used the functions “fitExtractVarPartModel” and “plotVarPart” from the R package variancePartition [12] to calculate and plot the variance of factors, using TMM-normalized data. All factors were modeled as random effects.

### Deconvolution with CIBERSORTx

For generation of cell type profiles used for deconvolution with CIBERSORTx, the bulk profiles were generated by inputting data sample-wise, TPM normalized, in a data matrix to CIBERSORTx. The reason for using TPM normalization was that this is recommended by the CIBERSORTx team and it was of interest to see the difference in performance between TPM and quantile normalization (TMM is expected to be in between given the normalization performance). The pooled single-cell profiles were handled in a similar way, where each pooled sample from the design matrix was treated as a single bulk sample. Profiles were created using only cells from datasets within the same lab. The final calculation of profiles was done by CIBERSORTx, using the “Create Signature Matrix” function (specifying RNA-Seq). For single-cell data, the cells of each type were sorted into 100 bins for disk quota reasons in CIBERSORTx. Each bin was given the gene expression of the mean of all the cells in the bin, TPM normalized. The final profiles were then generated using the CIBERSORTx “Create Signature Matrix” function (specifying sc RNA-Seq).

For generating mixtures used for deconvolution, the expression values of one T-cell sample multiplied by 0.5 was added to a similarly treated B-cell sample, both TMM normalized. The mixture samples were then TPM-normalized before being processed by CIBERSORTx. The reason for this procedure is that TPM-normalization before mixing the data could confound the mixture, creating a mixture where one cell type is given more weight, since library size normalization is sometimes inaccurate.

For the LM22 results, the fraction of B cells is the sum of the fractions for “B cells naive”, “B cells memory”, and “Plasma cells”. For the single-cell profile sets labeled “pooled” (7 and 8) cells were pooled and input to CIBERSORTx as bulk profiles. For the remaining single-cell profile sets (9–13) cells were pooled into 100 B cells and 100 T cells for each profile pair due to disk quota reasons and input as single-cell profiles. For evaluation of profiles generated from bulk data, the mixtures generated from the same lab as the profiles were removed to avoid using mixtures and profiles generated from the same data.

CIBERSORTx deconvolution was run using the function “Impute Cell Fractions”, specifying batch correction (B or S) and quantile normalization to produce such results. For quantile normalization, separate cell type profiles were produced, specifying that the cell type profiles should be quantile normalized.

CIBERSORTx was used from the CIBERSORTx web page (https://cibersortx.stanford.edu/), accessed 2020-06-05.

### Retrieval of transcript length and GC content

Transcript length was retrieved using the GenomicFeatures [51] R package (version 1.36.4) together with the biomaRt [52] package (version 2.40.0). We used the biomart ENSEMBL_MART_ENSEMBL (version 98) and the dataset mmusculus_gene_ensembl (version GRCm38.p6). We calculated GC content by using the R package BSgenome.Mmusculus.UCSC.mm10 [53] (version 1.4.0), together with GenomicFeatures and Biostrings [54].

### The UMICF covariate

The EVAL dataset contains both UMI counts and total counts, which makes it possible to calculate the number of copies per molecule. The UMICF covariate is calculated as

$$UMICF=\frac{total\;counts-UMI\;counts}{total\;counts}.$$

### Regressing out covariates

To regress out one or more covariates, a linear or loess (R package stats v3.6.1 using default parameter values) curve was first fitted to the log_2_ fold change between 10x and bulk in the covariates space. The curve was then regressed out of the 10x gene expression in log space as ***L_corr,i_ = L_orig,i_−p_i_+mean(p)***, where ***L***_***corr*,*i***_ is the corrected gene expression for gene ***i***, ***L***_***orig*,*i***_ is the original gene expression, ***p***_***i***_ is the predicted value of gene expression from the fit and ***mean(p)*** is the mean of all predicted values from the fit. The UMICF covariate was set to NA unless more than 5 unique UMIs were available for the gene, to avoid the noise induced by too few measurement points.

For all analyses, we removed a few outliers with extreme values for transcript length and GC content, in total 232 genes. UMICF was set to NA for all genes for which we had five or fewer UMIs (5321 and 6126 genes for cortex 1 and 2, respectively), since we deemed the amplification measure to be too noisy otherwise. All excluded genes were left untouched by the regression. In total (including genes for which UMICF was set to NA) 26,563 genes were used in the calculations for both Cortex 1 and Cortex 2. The genes where UMICF was set to NA were still used in the correlation calculation.

### Statistical tests

For estimating the confidence intervals in Fig 6, we bootstrapped the genes to include in the correlation for 10,000 iterations. The confidence intervals were then calculated as the values located at 2.5% and 97.5% in the sorted vector of correlations.

We used the function R function wilcox.test for the Wilcoxon signed rank tests for investigating if correlation differences between covariates were significant, with the parameters alternative = "greater", paired = TRUE. The same bootstraps were used for all covariates, making it possible to compare them with a paired test.

### Software

The data was analyzed using R version 3.6.1 and MATLAB R2018b. MATLAB was used for assembling the single-cell data and exporting the pooled samples to a text file; the rest of the analysis was done in R. The MATLAB code uses the component SingleCellToolbox for importing public single-cell datasets (https://github.com/SysBioChalmers/SingleCellToolbox). The processed data and source code are available at: http://doi.org/10.5281/zenodo.4011593. To ensure the quality of our analyses, we verified and validated the code using a combination of test cases, reasoning around expected outcome of a function and code review. The details of this activity are available in the verification matrix available with the code.

## Supporting information

S1 Fig PCA of batch corrected data using ComBat where cell type is not specified in the design matrix. (PDF)

S2 Fig Explained variance per gene expression for the LM22 geneset across all bulk samples. The plot shows how the explained variance by the different factors change with gene expression (Loess fit, span = 0.3).
(PDF)

S3 Fig Explained variance in gene expression for bulk RNA-seq samples using batch corrected data. A. All genes (12072 genes). B. Housekeeping genes (3393 genes). C. LM22 (395 genes). D. LM22S genes. E. Identical to D, with the difference that cell subtype is replaced with cell type (B/T). F. Explained variance per gene expression. The plot shows how the explained variance by the different factors change with gene expression (Loess fit, span = 0.3).
(PDF)

S4 Fig Additional plots for explained variance. A, B: Comparison between TMM and Quantile Normalization. C-D: The effect of including Smart-Seq2 samples. E. Variance explained by having samples from different individuals compared to samples from the same individual but taken at different time points. F. Variance explained by having different samples compared to technical replicates, where the same sample has been sequenced several times. G, H. Same data as E, but separated on cell type into two groups to make individual factor more comparable to the technical replicates shown in F.
(PDF)

S5 Fig Average gene expression per gene vs the UMICF covariate. The figure presents data from the EVAL dataset, Cortex 1, 10x single-cell data, normalized using TMM. Only genes with 5 molecules or more is shown.
(PDF)

S6 Fig Version of main Fig 6 calculated on quantile normalized data. A. Gene expression for cortex 1 from the EVAL dataset plotted as 10x vs bulk. The red line represents a perfect correlation. B. Gene expression for cortex 1 from the EVAL dataset after regressing out the differences in UMICF and GC content between 10x and bulk using a loess fit, which improves the correlation. C. Average Pearson correlation coefficient between 10x data and bulk in log scale after regressing out technical covariates (UMI copy fraction, transcript length, GC content and GC content tail), using linear or loess regression. The correlation shown is the average of the correlations from cortex 1 and 2 of the EVAL dataset, using quantile normalization.
(PDF)

S1 Table Sample Information. (XLSX)

S2 Table The number of cells used for each single-cell profile pair used in Fig 7 in the main text. (PDF)

S1 Note The role of sampling effects when regressing out the UMICF variable. (PDF)
