# Supplementary material for: Sources of variation in cell-type RNA-Seq profiles
Source: PLoS One. 2020 Sep 21;15(9):e0239495. doi: 10.1371/journal.pone.0239495 (PMC7505444; doi:10.1371/journal.pone.0239495)
Supplement: S1 Fig — (PDF) [file pone.0239495.s001.pdf]

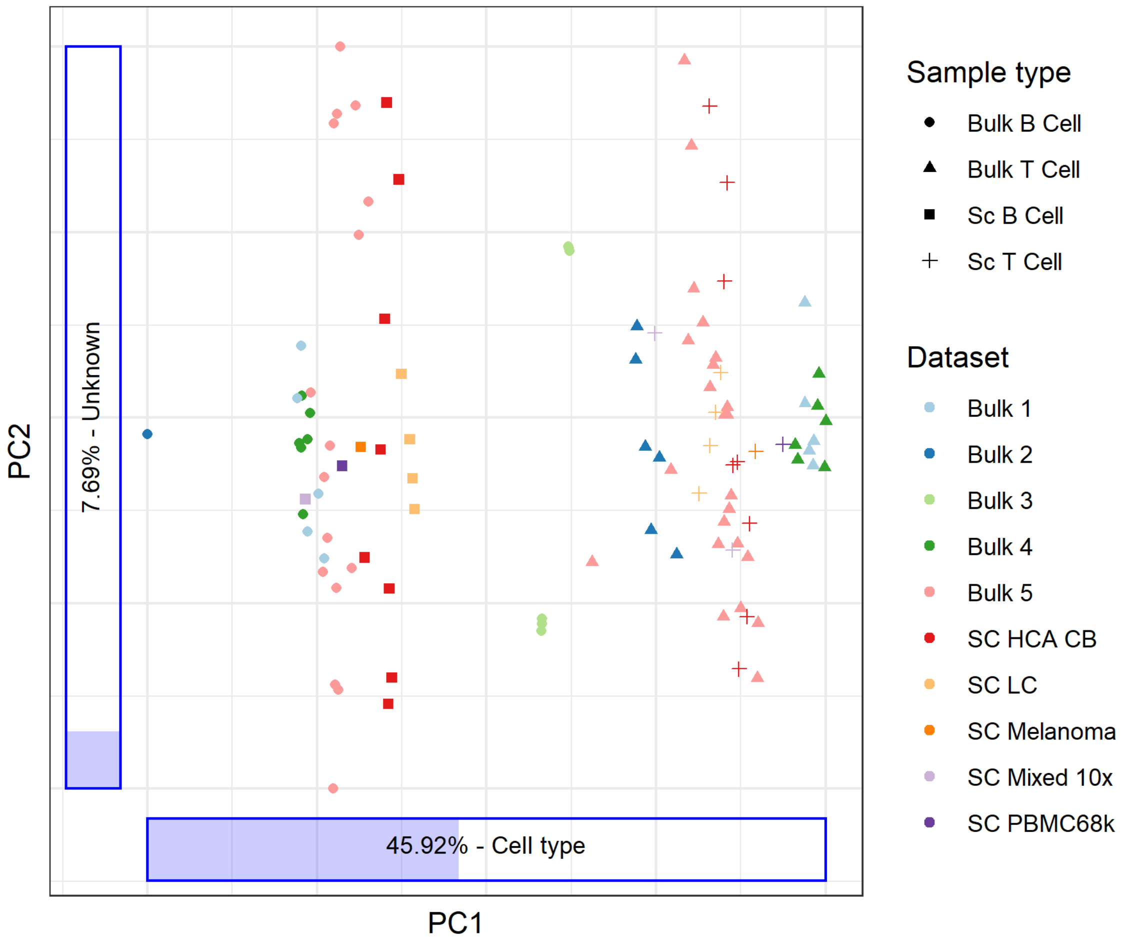

S1 Fig. PCA of batch corrected data using ComBat where cell type is not specified in the design matrix.
