# Supplementary material for: Sources of variation in cell-type RNA-Seq profiles
Source: PLoS One. 2020 Sep 21;15(9):e0239495. doi: 10.1371/journal.pone.0239495 (PMC7505444; doi:10.1371/journal.pone.0239495)
Supplement: S2 Fig — The plot shows how the explained variance by the different factors change with gene expression (Loess fit, span = 0.3). (PDF) [file pone.0239495.s002.pdf]

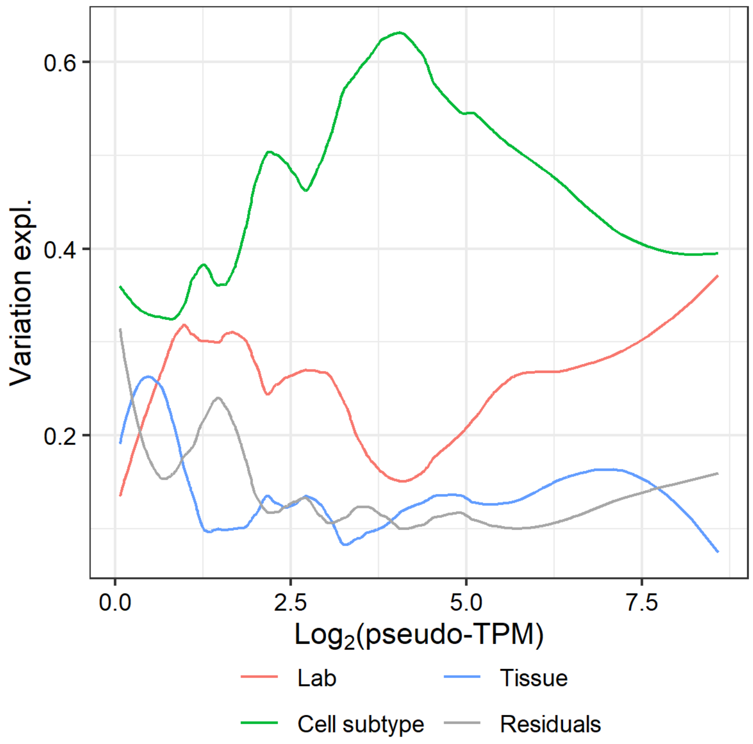

S2 Fig. Explained variance per gene expression for the LM22 geneset across all bulk samples. *The plot shows how the explained variance by the different factors change with gene expression (Loess fit, span = 0.3).*
