# Supplementary material for: Sources of variation in cell-type RNA-Seq profiles
Source: PLoS One. 2020 Sep 21;15(9):e0239495. doi: 10.1371/journal.pone.0239495 (PMC7505444; doi:10.1371/journal.pone.0239495)
Supplement: S3 Fig — A. All genes (12072 genes). B. Housekeeping genes (3393 genes). C. LM22 (395 genes). D. LM22S genes. E. Identical to D, with the difference that cell subtype is replaced with cell type (B/T). F. Explained variance per gene expression. The plot shows how the explained variance by the different factors change with gene expression (Loess fit, span = 0.3). (PDF) [file pone.0239495.s003.pdf]

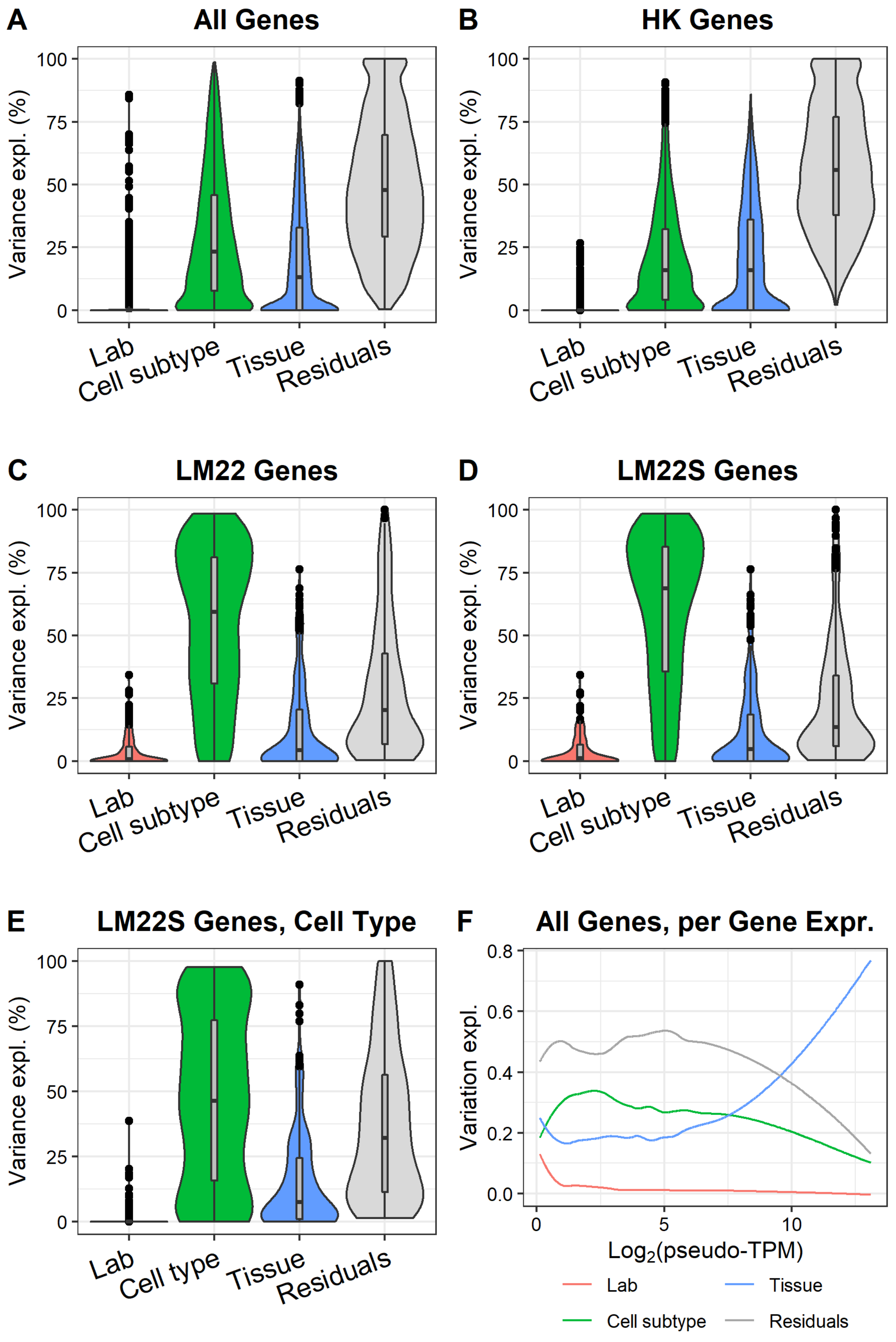

S3 Fig. Explained variance in gene expression for bulk RNA-seq samples using batch corrected data. *A. All genes (12072 genes). B. Housekeeping genes (3393 genes). C. LM22 (395 genes). D. LM22S genes. E. Identical to D, with the difference that cell subtype is replaced with cell type (B/T). F. Explained variance per gene expression. The plot shows how the explained variance by the different factors change with gene expression (Loess fit, span = 0.3).*
