# Supplementary material for: Sources of variation in cell-type RNA-Seq profiles
Source: PLoS One. 2020 Sep 21;15(9):e0239495. doi: 10.1371/journal.pone.0239495 (PMC7505444; doi:10.1371/journal.pone.0239495)
Supplement: S4 Fig — A, B: Comparison between TMM and Quantile Normalization. C-D: The effect of including Smart-Seq2 samples. E. Variance explained by having samples from different individuals compared to samples from the same individual but taken at different time points. F. Variance explained by having different samples compared to technical replicates, where the same sample has been sequenced several times. G, H. Same data as E, but separated on cell type into two groups to make individual factor more comparable to the technical replicates shown in F. (PDF) [file pone.0239495.s004.pdf]

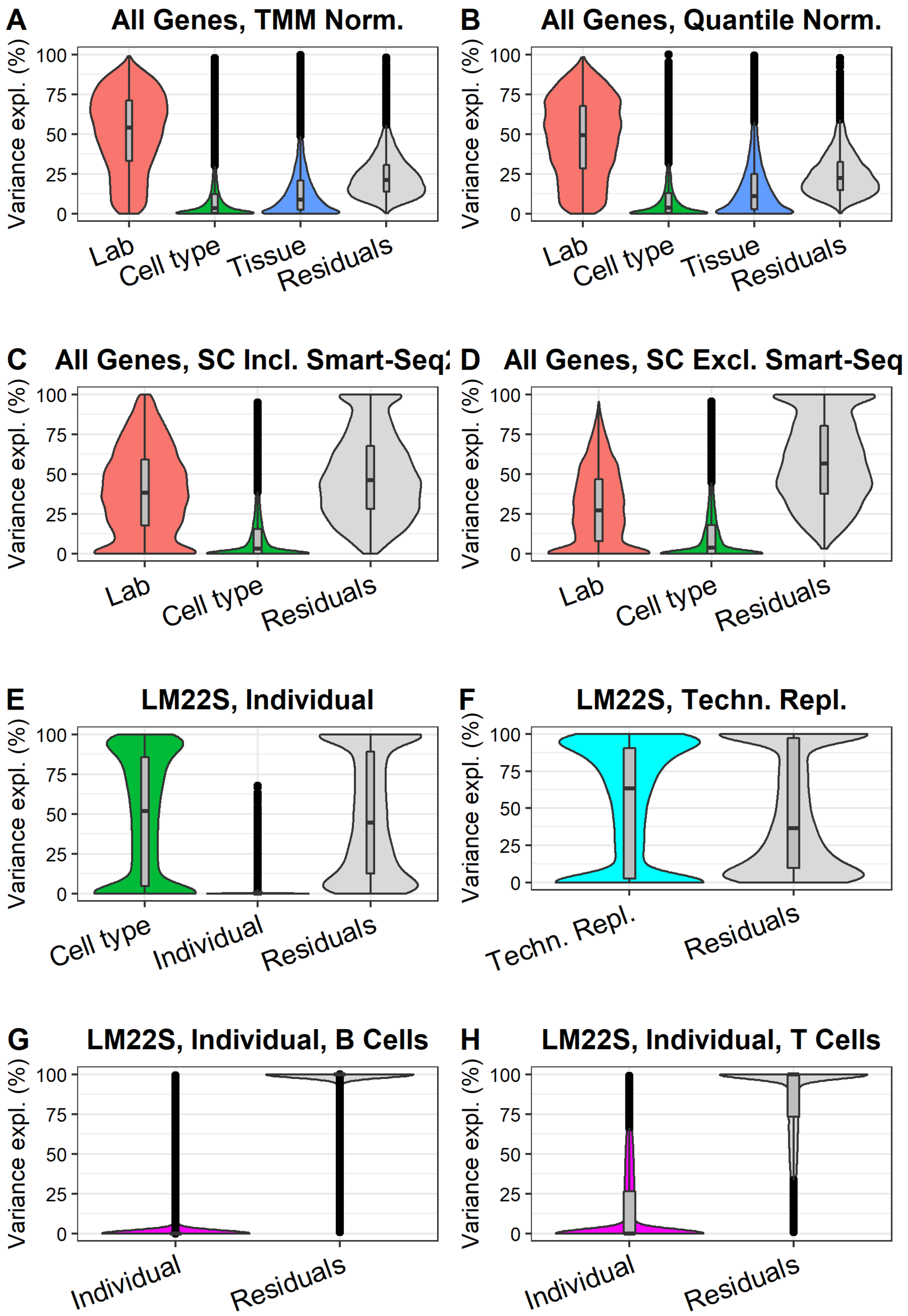

Fig. S4. Additional plots for explained variance. *A, B: Comparison between TMM and Quantile Normalization. C-D: The effect of including Smart-Seq2 samples. E. Variance explained by having samples from different individuals compared to samples from the same individual but taken at different time points. F. Variance explained by having different samples compared to technical replicates, where the same sample has been sequenced several times. G, H. Same data as E, but separated on cell type into two groups to make individual factor more comparable to the technical replicates shown in F.*
