# Supplementary material for: Sources of variation in cell-type RNA-Seq profiles
Source: PLoS One. 2020 Sep 21;15(9):e0239495. doi: 10.1371/journal.pone.0239495 (PMC7505444; doi:10.1371/journal.pone.0239495)
Supplement: S5 Fig — The figure presents data from the EVAL dataset, Cortex 1, 10x single-cell data, normalized using TMM. Only genes with 5 molecules or more is shown. (PDF) [file pone.0239495.s005.pdf]

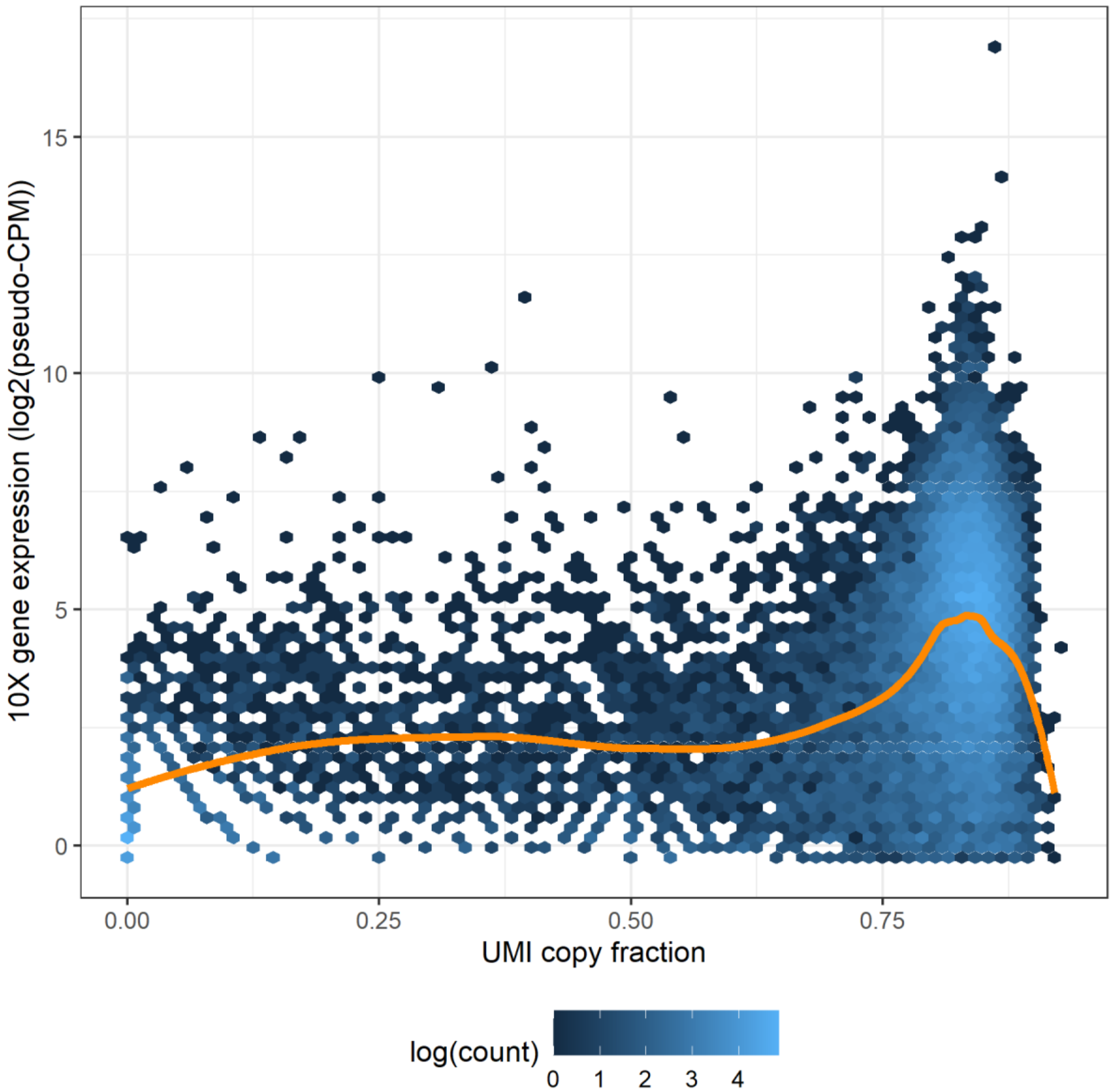

Fig. S5. Average gene expression per gene vs the UMICF covariate. *The figure presents data from the EVAL dataset, Cortex 1, 10x single-cell data, normalized using TMM. Only genes with 5 molecules or more is shown.*
