# Supplementary material for: Sources of variation in cell-type RNA-Seq profiles
Source: PLoS One. 2020 Sep 21;15(9):e0239495. doi: 10.1371/journal.pone.0239495 (PMC7505444; doi:10.1371/journal.pone.0239495)
Supplement: S6 Fig — A. Gene expression for cortex 1 from the EVAL dataset plotted as 10x vs bulk. The red line represents a perfect correlation. B. Gene expression for cortex 1 from the EVAL dataset after regressing out the differences in UMICF and GC content between 10x and bulk using a loess fit, which improves the correlation. C. Average Pearson correlation coefficient between 10x data and bulk in log scale after regressing out technical covariates (UMI copy fraction, transcript length, GC content and GC content tail), using linear or loess regression. The correlation shown is the average of the correlations from cortex 1 and 2 of the EVAL dataset, using quantile normalization. (PDF) [file pone.0239495.s006.pdf]

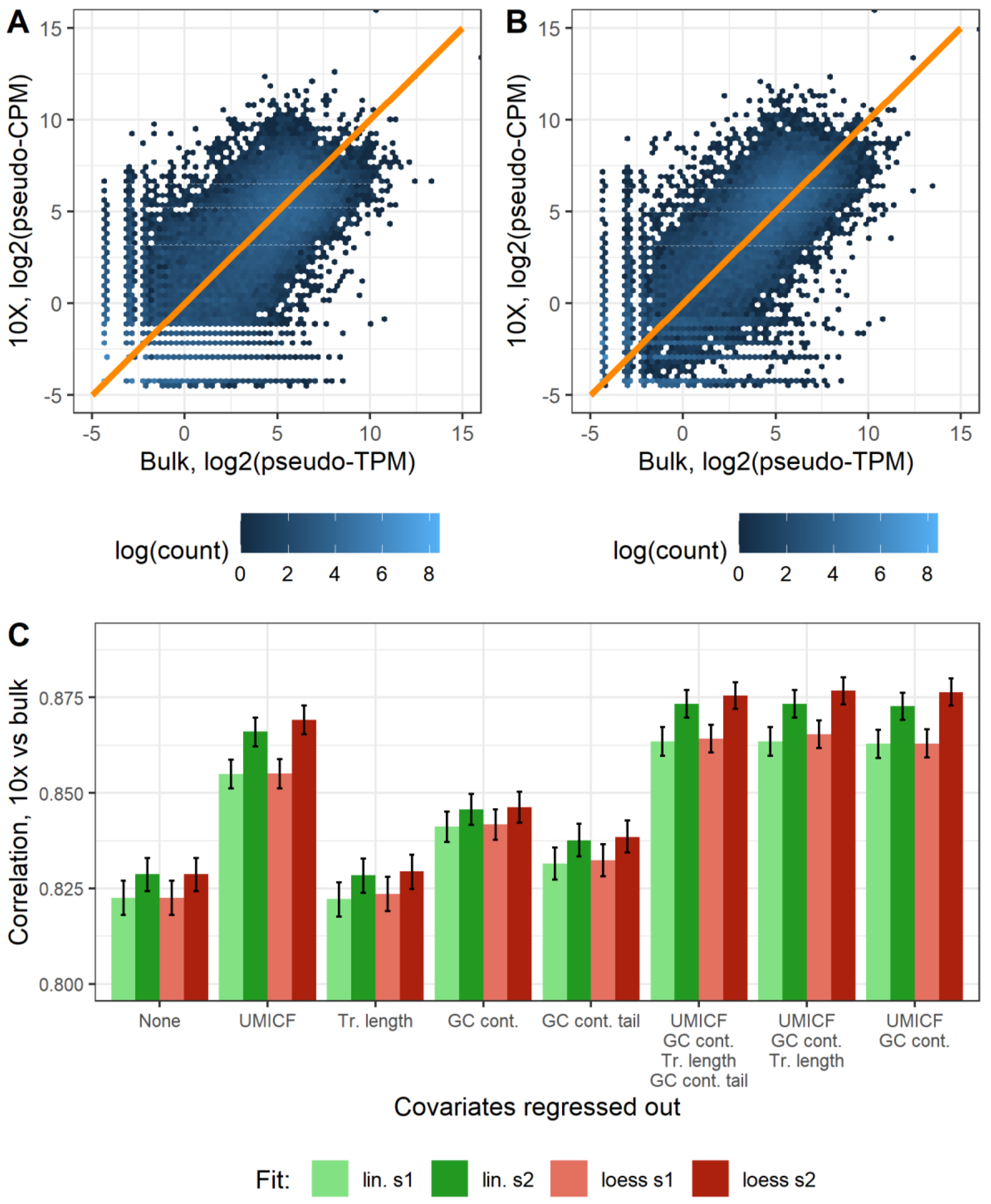

S6 Fig. Version of main figure 6 calculated on quantile normalized data. *A. Gene expression for cortex 1 from the EVAL dataset plotted as 10x vs bulk. The red line represents a perfect correlation. B. Gene expression for cortex 1 from the EVAL dataset after regressing out the differences in UMICF and GC content between 10x and bulk using a loess fit, which improves the correlation. C. Average Pearson correlation coefficient between 10x data and bulk in log scale after regressing out technical covariates (UMI copy fraction, transcript length, GC content and GC content tail), using linear or loess regression. The correlation shown is the average of the correlations from cortex 1 and 2 of the EVAL dataset, using quantile normalization.*
