# Supplementary material for: Sources of variation in cell-type RNA-Seq profiles
Source: PLoS One. 2020 Sep 21;15(9):e0239495. doi: 10.1371/journal.pone.0239495 (PMC7505444; doi:10.1371/journal.pone.0239495)
Supplement: S2 Table — (PDF) [file pone.0239495.s008.pdf]

***S2 Table.*** *The number of cells used for each single-cell profile pair used in figure 7 in the main text.*

| **Profile** | **No. B Cells** | **No. T Cells** |
|---|---|---|
| Pooled SC HCA CB | 35,910 | 167,612 |
| Pooled SC LC | 4,806 | 23,170 |
| SC PBMC68k | 5,908 | 48,657 |
| SC Melanoma | 512 | 2,040 |
| SC LC Tumor | 4,509 | 18,306 |
| SC LC Healthy Tissue | 297 | 4,864 |
| SC HCA CB | 35,910 | 167,612 |
