# Supplementary material for: Sources of variation in cell-type RNA-Seq profiles
Source: PLoS One. 2020 Sep 21;15(9):e0239495. doi: 10.1371/journal.pone.0239495 (PMC7505444; doi:10.1371/journal.pone.0239495)
Supplement: S1 Note — (PDF) [file pone.0239495.s009.pdf]

Supporting note 1 - The role of sampling effects when regressing out the UMICF variable

The UMI copy fraction covariate used in the main text is correlated with gene expression, which could partly be explained by sampling effects on lowly expressed genes (S5 Fig). However, there are also many genes with high expression and low UMICF, ruling out the possibility that this effect has its origin in sampling effects only. Since the UMICF variable is correlated with gene expression, we investigated if that could explain the improvement in correlation between single-cell and bulk RNA-Seq from regressing out the UMICF variable. We reasoned that systematic differences in gene expression over the gene expression range between single-cell and bulk could indirectly be regressed out when regressing out UMICF and consequently make the sample data more similar. If this would be the case, regressing out UMICF could be replaced by quantile normalization, which effectively removes any such differences. To test this, we performed the same analysis as in the main text (i.e. comparing 10x single-cell data with bulk from the EVAL dataset) but using quantile normalization instead of TMM (S6 Fig). Compared to the case for TMM normalization, regressing out the UMICF covariate is slightly less effective, but the results are still similar. This suggests that systematic differences in gene expression over the gene expression range between single-cell and bulk can only explain a small part of the improvement in correlation of gene expression between single-cell and bulk resulting from regressing out the UMICF covariate.
